# Exenatide Once Weekly in the Treatment of Patients with Multiple System Atrophy

**DOI:** 10.1002/ana.70004

**Published:** 2025-07-29

**Authors:** Nirosen Vijiaratnam, Christine Girges, Martin Wiegand, Claudia Ismail, Alexandra Lameirinhas, Alison Yarnall, Cameron Kirk, Silvia Del‐Din, Lynn Rochester, Christopher Kobylecki, Gareth Ambler, Simon Skene, Henry Houlden, Viorica Chelban, Amanda Heslegrave, Wendy Phillips, Alan Whone, Niall Quinn, Christian Lambert, Charlotte Dore, Huw R. Morris, Mathew H. Horrocks, Ji Eun Lee, Judi O'Shaughnessy, Yazhou Li, Nigel H. Greig, Sonia Gandhi, Vincenzo Libri, Dilan Athauda, Tom Foltynie

**Affiliations:** ^1^ Department of Clinical and Movement Neurosciences University College London Queen Square Institute of Neurology London UK; ^2^ National Hospital for Neurology and Neurosurgery London UK; ^3^ Department of Statistical Science, University College London London UK; ^4^ NIHR UCLH Clinical Research Facility London England UK; ^5^ Newcastle NIHR BRC, Translational and Clinical Research Institute Newcastle University, Newcastle Upon Tyne England UK; ^6^ Manchester Centre for Clinical Neurosciences, Northern Care Alliance NHS Foundation Trust, Manchester Academic Health Science Centre University of Manchester Manchester UK; ^7^ Surrey Clinical Trials Unit University of Surrey. Guildford Guildford UK; ^8^ Department of Neuromuscular Diseases, Queen Square Institute of Neurology University College London London England UK; ^9^ University College London, UCL UKDRI, Dept of Neurodegenerative Diseases, Queen Square House London England UK; ^10^ Cambridge University Hospitals NHS Foundation Trust Cambridge UK; ^11^ Movement Disorders Group, Bristol Brain Centre, Southmead Hospital Bristol UK; ^12^ Functional Imaging Laboratory, Department of Imaging Neuroscience London England UK; ^13^ EaStCHEM School of Chemistry The University of Edinburgh Edinburgh UK; ^14^ IRR Chemistry Hub, Institute for Regeneration and Repair The University of Edinburgh Edinburgh UK; ^15^ Intramural Research Program, National Institute on Aging National Institutes of Health Baltimore MD USA; ^16^ The Francis Crick Institute London England UK

## Abstract

**Objective:**

Exenatide, a glucagon‐like peptide‐1 (GLP‐1) receptor agonist, has neuroprotective effects in preclinical models of multiple system atrophy (MSA). We investigated these effects in a proof‐of‐concept clinical trial.

**Methods:**

In this single‐center, randomized, open label trial, participants with MSA were randomly assigned (1:1) to receive subcutaneous injections of exenatide 2 mg weekly for 48 weeks, or as controls, followed by a 48‐week washout period. The primary outcome was the Unified Multiple System Atrophy Rating Scale (UMSARS) parts I + II combined score at 48 weeks. Objective secondary outcome measures included the numbers of participants losing ambulation; scoring ≥ 3 on UMSARS part I items for falls, speech, swallowing, as well as timed walking and measures of quality of life and cognition.

**Results:**

Between September 23, 2020, and May 6, 2022, 50 participants were recruited (25 in each group). At 48 weeks, UMSARS parts I + II scores had worsened by 6.1 points (95% confidence interval [CI] = 3.0 to 9.3, SD = 6.9) in the exenatide group and by 13 3 points (95% CI = 9.2 to 17.3, SD = 9.4) in the control group, an adjusted mean difference of −7.4 points (−11.3 to −3.6, *p* = 0.0003). There were no statistically significant differences at either 48 or 96 weeks in the secondary outcome measures. Biomarker analysis of neurofilament light chain and cerebral spinal fluid (CSF) alpha‐synuclein oligomer load, sensor‐derived gait measures, and imaging findings were also similar between groups.

**Interpretation:**

Exenatide was associated with positive effects on participant‐reported symptoms and clinician‐rated MSA severity. In contrast, none of the objective comparisons differed according to randomization. Given the open label trial design, the discrepancy between the primary outcome and the objective measures may be explicable as placebo effects/observer bias. ANN NEUROL 2025;98:991–1003

Multiple system atrophy (MSA) is a rare, progressive neurodegenerative disorder characterized pathologically by glial cytoplasmic inclusions which are composed of alpha‐synuclein. Patients present with a combination of autonomic dysfunction, parkinsonism, and cerebellar impairment. No effective approaches for slowing disease progression currently exist and survival ranges between 6 and 9 years from diagnosis.^1^ There is a great unmet need for therapies which modify the rate of progression of this disease.

Type 2 diabetes mellitus (T2DM) is a risk factor for developing neurodegenerative diseases such as Parkinson's disease (PD), whereas patients with both PD and T2DM have a more aggressive rate of PD progression.^2^, ^3^, ^4^ Shared dysfunction in insulin signaling may link diabetes and neurodegeneration. Insulin receptors are found throughout the brain and insulin is responsible for modulating many cellular processes, including inflammation, protein aggregation, oxidative stress, autophagy, and apoptosis, via the actions of 2 main downstream pathways; Akt and mitogen activated protein kinase (MAPK).^5^, ^6^ Dysregulation of these processes are also thought to be partly involved in the pathogenesis of MSA.^7^

Peripheral insulin and insulin‐like growth factor (IGF‐1) levels are increased in the serum of patients with MSA, and correlate with disease progression.^8^ Furthermore, increased expression of insulin resistance markers in the context of neuronal cell loss has also been noted in the brains of patients with MSA compared with healthy controls.^9^ Modulating insulin/IGF‐1 signaling might therefore provide an effective approach to disease modification.

Exenatide is a glucagon‐like peptide‐1 receptor agonist (GLP‐1 RA) and was the first of multiple GLP‐1 RAs, which have been used to treat T2DM since 2005. The GLP‐1 receptor is expressed throughout the brain and influences the insulin sensitivity of neuronal and glial cells.^10^ Activation of this receptor by exenatide in animal models has shown protective effects in neurodegenerative diseases through a positive impact on cell survival, apoptosis, and protein aggregation.^6^, ^11^ This has been reinforced by positive findings in 2 clinical trials of exenatide in participants with moderate stage PD showing potential disease modifying effects.^12^, ^13^, ^14^, ^15^ A further study, using exenatide (via subcutaneous infusion) in a transgenic mouse model of MSA, showed positive effects on insulin resistance and monomeric alpha‐synuclein levels in the striatum, as well as improving survival of nigral dopamine neurons.^9^

Based on these preclinical findings and earlier encouraging results of clinical trials in PD, we designed a proof‐of‐concept trial to assess the potential use of exenatide as a disease‐modifying treatment for MSA. The trial was investigator initiated and had to be configured as an open label trial due to the non‐availability of placebo pen injections from the manufacturer and the complexity and cost of the pen injection device preventing placebo supplies from other sources.

## Methods

### Study Design

We conducted a randomized, phase IIa, open label, parallel‐group, single‐center trial of exenatide once weekly in MSA. Participants were recruited at the Leonard Wolfson Experimental Neuroscience Centre (London, UK), a dedicated research facility and part of the University College London (UCL) Institute of Neurology and the National Hospital for Neurology and Neurosurgery. The study was coordinated by the UCL Joint Research Office. Clinical oversight was provided by a trial steering committee and an independent data and safety monitoring board. The trial was approved by the London‐Hampstead Research Ethics Committee (reference 20/LO/0473). All participants provided written informed consent. The trial was registered with clinicaltrials.gov (NCT04431713).

### Participants

Eligible men and women were aged 30 to 80 years and had a diagnosis of possible or probable MSA of the parkinsonian (MSA‐P) or cerebellar subtype (MSA‐C) according to the Gilman Criteria.^16^ Participants were less than 5 years from the time of documented MSA diagnosis, or from the time of previously documented parkinsonian/ataxic neurological condition, which was later re‐diagnosed as MSA. They were able to walk at least 10 meters (with or without assistance) and had an anticipated survival of at least 3 years in the opinion of the investigator.

Exclusion criteria were concurrent dementia (defined as a Montreal Cognitive Assessment [MoCA] score < 21), body mass index (BMI) < 18.5, glycated hemoglobin (HbA1c) ≥ 48 mmol/mol, or random blood glucose >11.1 mmol/L at screening, which may indicate T2DM, previous exposure to exenatide within preceding 90 days, concurrent severe depression (Beck Depression Inventory II [BDI‐II] ≥ 30), active malignancy, or other clinically significant medical diagnosis. Participants who met any of the following criteria, which suggested advanced disease, were also excluded due to speech impairment, as assessed by a score of ≥ 3 on the Unified Multiple System Atrophy Rating Scale (UMSARS) question 1, swallowing impairment, as assessed by a score of ≥ 3 on UMSARS question 2, impairment in ambulation, as assessed by a score of >3 on UMSARS question 7, and falling more frequently than once per week, as assessed by a score of ≥ 3 on UMSARS question 8.

### Randomization

Eligible participants were randomized using an independent internet‐based service (Sealed Envelope). Stratified randomization was used with varying block sizes and 2 strata according to disease subtype (MSA‐P vs MSA‐C). Participants were randomly assigned (1:1) to self‐administer subcutaneous exenatide in the form of Bydureon or Bydureon BCise at a dose of 2 mg once weekly in addition to their standard of care, or to continue standard of care alone.

### Procedures

The trial had a washout design, comprising a 48‐week treatment exposure period. After completion of all baseline assessments, participants randomized to exenatide were instructed on how to administer the injections and were witnessed administering their first injection. Participants underwent assessments at baseline, and weeks 12, 24, 36, and 48, and then underwent a final assessment after a prolonged drug washout period at week 96.

Participants who were taking dopaminergic medication were asked to attend each visit in an off‐medication state if they were able to tolerate this. This was defined as a period of withdrawal of levodopa for at least 8 hours (ie, overnight) or 36 hours in the case of long‐acting drugs (eg, ropinirole, pramipexole, rasagiline, and rotigotine). The UMSARS and a timed 10‐minute sit‐stand‐walk test were performed at each visit (the latter was also repeated 1 hour after dopaminergic medications in the subset of individuals who were able to stop dopaminergic medication). A single rater who was an experienced neurologist in movement disorders performed the UMSARS for all participants. Additional assessments comprised the UMSARS part IV (global disability scale), MoCA, MSA‐Quality of Life (MSA‐QOL) scale, BDI‐II, Unified Dystonia Rating Scale, and Clinician Global Impression of Change (CGI).

Digital measures of gait (including step count, walking speed, and stride length) were collected over a weeklong period prior to baseline and at 48 weeks via a CE marked sensor (Axivity)^17^ attached to the lower back. Blood was collected at each visit, and cerebrospinal fluid (CSF) was collected at baseline and 48 weeks and stored in aliquots at −80°C.

At each visit, adverse events, biochemical results, and vital signs (supine and standing) were recorded. Injection diaries were collected to assess compliance. Concomitant medications were systematically documented, and adjustments of symptomatic treatments were permitted throughout the trial.

### Outcomes

The primary outcome was the UMSARS (parts I and II combined score) at 48 weeks comparing exenatide to best medically treated participants. Due to the open label nature of the trial, it was impossible to adequately blind the assessing neurologist to the randomization outcome. Predefined secondary outcomes comprised the following measures at 48 and 96 weeks; loss of independent ambulation, defined by a score of 4 in UMSARS part I item 7 (walking); timed walk; MSA‐QOL scores; UMSARS parts I, II, III, and IV scores; anti‐parkinsonian or anti‐orthostatic hypotension medication use; number of falls; and the proportion of participants reaching a score of ≥ 3 on UMSARS part I items 1 (speech), 2 (swallowing), and 8 (falling); CGI (based on ordinal scale 0–7); and MoCA scores. Exploratory outcomes included changes in digital mobility outcomes, as measured by the Axivity device,^18^ changes in grey matter/white matter volume on magnetic resonance imaging (MRI), plasma and CSF neurofilament light (NfL) levels, and CSF alpha‐synuclein oligomer levels between baseline and 48 weeks.

The digital mobility outcomes were estimated from data captured using the Axivity AX6 device (York, UK) which was worn on the lower back using a custom designed adhesive patch for 7 consecutive days, with individuals asked to continue their normal weekly routine. The validated Mobilise‐D processing pipeline^19^, ^20^, ^21^ was used to identify walking bouts and extract gait metrics to calculate selected digital mobility outcomes in this study; daily step count, walking speed, and stride length. Daily step count was estimated from all real‐world walking combined, where walking speed and stride length were estimated from only short bouts of walking that take place between 10 and 30 seconds.

A subgroup of participants were invited to have an MRI (N = 32 baseline, N = 14 at week 48 with 7 on treatment and 7 controls). All MRIs were acquired on a 3 T whole‐body MRI system (MAGNETOM Prisma‐fit; Siemens Healthcare, Erlangen, Germany) using a 64‐channel head coil. The acquisition included an 800‐micron, isotropic multi‐parameter mapping (MPM) protocol,^22^ The hMRI toolbox was used to reconstruct quantitative magnetization transfer, proton density, R1, and R2*, changing the default tissue probability maps to brain‐spine priors^23^ to provide better coverage of the caudal brainstem and upper cervical cord. All images were rigidly aligned to an MNI orientated brain. A 2‐pass warping step was performed. Initially, the geodesic “Shoot” toolbox^24^ in SPM was used to generate an initial group average template from the output of SPM segment using the brain‐spine priors (grey matter, white matter, and CSF). Total intracranial volume was also calculated from these initial segmentations using the SPM “Tissue Volumes” function. The second step used updated brainstem and cortical tissue priors^25^ to provide a more detailed segmentation (8 tissue classes based on the magnetization transfer and proton density images), and these were used to estimate the final group average template. Warped modulated tissue segmentations were generated using the push‐forward function in the SPM deformations toolbox. Difference images between baseline and 48 weeks were calculated (ie, rate of tissue change per year). Two levels of Gaussian spatial smoothing was applied, 2 mm full‐width half maximum for brainstem analyses (to account for the small nuclei sizes in this region)^26^ and 6 mm for the rest of the brain.

Assessment of serum and CSF concentrations of exenatide was done in a blinded manner in duplicate across all samples by fluorescent enzyme‐linked immunosorbent assay (ELISA) immunoassay (FEK‐070‐94; Phoenix Pharmaceuticals, Burlingame, CA) at the National Institute on Aging (Baltimore, MD).

Plasma and CSF NfL concentrations were measured using the 1‐plex Single molecule array (Simoa) kit (NF‐Light, Quanterix) on a Simoa HD‐1 Analyzer (Quanterix, Billerica, MA)^27^ according to the manufacturer's instructions. Samples were tested in a blinded manner at the UCL biomarkers laboratory using one batch of reagents. All NfL values were within the linear range of the assay.

Assessment of oligomer levels in CSF was performed using single aggregate visualization by enhancement (SAVE) imaging, which has been validated in other alpha‐synucleinopathies such as PD.^28^ It utilizes the amyloid‐binding dye thioflavin‐T (ThT) with total internal reflection fluorescence (TIRF) microscopy to visualize and quantify oligomers at the single‐molecule level. Borosilicate glass cover slips (22 × 40 mm, VWR = 6310135) were subjected to an argon plasma (Zepto, Diener, Germany) for 1 hour to remove any fluorescent residues. Frame‐Seal slide chambers (9 × 9 mm,^2^; Biorad, Hercules, CA; SLF‐0601) were attached to the glass, and 70 μL of poly‐L‐lysine (70 k‐150 k molecular weight; Sigma‐Aldrich; 25,988–63‐0) were added to the chamber and incubated for at least 30 minutes, before washing with 0.02 μm filtered phosphate‐buffered saline (PBS; pH 7.4). CSF samples were diluted 10‐fold in 0.02 μm filtered PBS and added to the chamber and incubated for 1 hour. The sample was removed and the surface was washed 3 times with filtered PBS, before 5 μM ThT (Abcam; ab120751) was added. Imaging was carried out on a custom‐built TIRF microscope, described previously,^29^ using 405 nm excitation (approximately 100 W cm^−2^). Images were collected with an exposure time of 50 ms for a total of 50 frames. To reduce region bias, 25 images were collected spaced 200 μm apart, for 3 separate regions of the slide.

### Statistical Analysis

Based on a trial investigating rasagiline versus placebo for MSA (MSA‐Ras) using the UMSARS total score (parts I and II) as the primary efficacy measure, demonstrating a mean 7.8 points decline in the placebo group (SD = 10.4) after 48 weeks of treatment,^30^ we estimated that a sample size of 40 participants (20 per group) would be required to detect a difference of 5 total UMSARS points between the 2 groups, assuming a correlation between baseline and final disease severity of 0.85 with a 2‐sided 5% significance level. These calculations were based on a common SD of 10.4, 80% power, and an overall type 1 error rate of 5%. A 5‐point difference between groups would be a clinically important effect size. Based on a 20% dropout rate, as reported from previous trials, the sample size was chosen to be 50 (25 per arm).

All study analyses were done according to a predefined statistical analysis plan. The main statistical analysis of the primary end point was based on the intention‐to‐treat population (ITT). The primary analysis compared exenatide participants to best medically treated participants using a 2‐level mixed model that included the total UMSARS scores from 4 timepoints (12 weeks, 24 weeks, 36 weeks, and 48 weeks) with interaction terms between the intervention and timepoint indicator variables to enable estimation of the effect of intervention at 48 weeks. This model also adjusted for baseline UMSARS score and MSA subtype using fixed effects. Data were included from all participants with at least 1 follow up timepoint. We ran a variety of sensitivity analyses after imputing the follow‐up data under assumptions of both MAR (missing at random) and NMAR (not missing at random).^31^ Differences in secondary outcomes were estimated with the same approach. We used Cox proportional hazard models to compare the time to specific milestones at any point before week 48. Breslow's method was used to correct for ties and results were checked using exact methods. Ordinal logistic regression was used to analyze the secondary outcomes CGI and UMSARS part IV.

Comparison of adverse events between treatment groups was done with χ^2^ tests. Comparisons of changes in biomarker data between baseline and 48 weeks according to randomization allocation used Kruskal Wallis tests. A complier average causal effect analysis (CACE) was also prespecified, which estimates the effect of treatment (via 2‐stage least squares) for patients that comply with the protocol (eg, abstention from exenatide if randomized to control). All assumptions were deemed plausible on theoretical grounds.^32^

The CSF oligomer data were analyzed using custom‐written code in Python 3.8 (code available at 10.5281/zenodo.7546532). The images were first averaged over the 20 frames, and the background subtracted using threshold local in skimage filters. Spots corresponding to ThT‐bound aggregates were selected by applying a threshold equal to the mean + 5 × SD of the intensity in each image.

For the digital mobility outcomes, changes between baseline and 48 weeks were calculated and compared using appropriate parametric (*t* test) or non‐parametric (Mann–Whitney test) statistics.

MRI scan data were compared in 2 ways using voxel‐based morphometry (VBM). The first used the difference images, which reflected the relative rate of tissue volume change, and the second contrasted the 48 week scans. Age, biological sex, and baseline total intracranial volume were used as covariates. All warped‐modulated tissue was contrasted using a 2‐sample *t* test (both classic SPM and also the updated tissue priors designed to provide more accurate alignment particularly in the brainstem) resulting in 40 SPM contrasts total. Each contrast was masked using the corresponding group‐average tissue class thresholded at a probability of 0.2. Peak family‐wise error corrected *p* < 0.05 was considered significant but given the small sample size all results were also viewed at *p* < 0.001 uncorrected to look for changes in a priori basal ganglia regions, and a small‐volume analysis was also performed in the substantia nigra.

## Results

**FIGURE 1:**
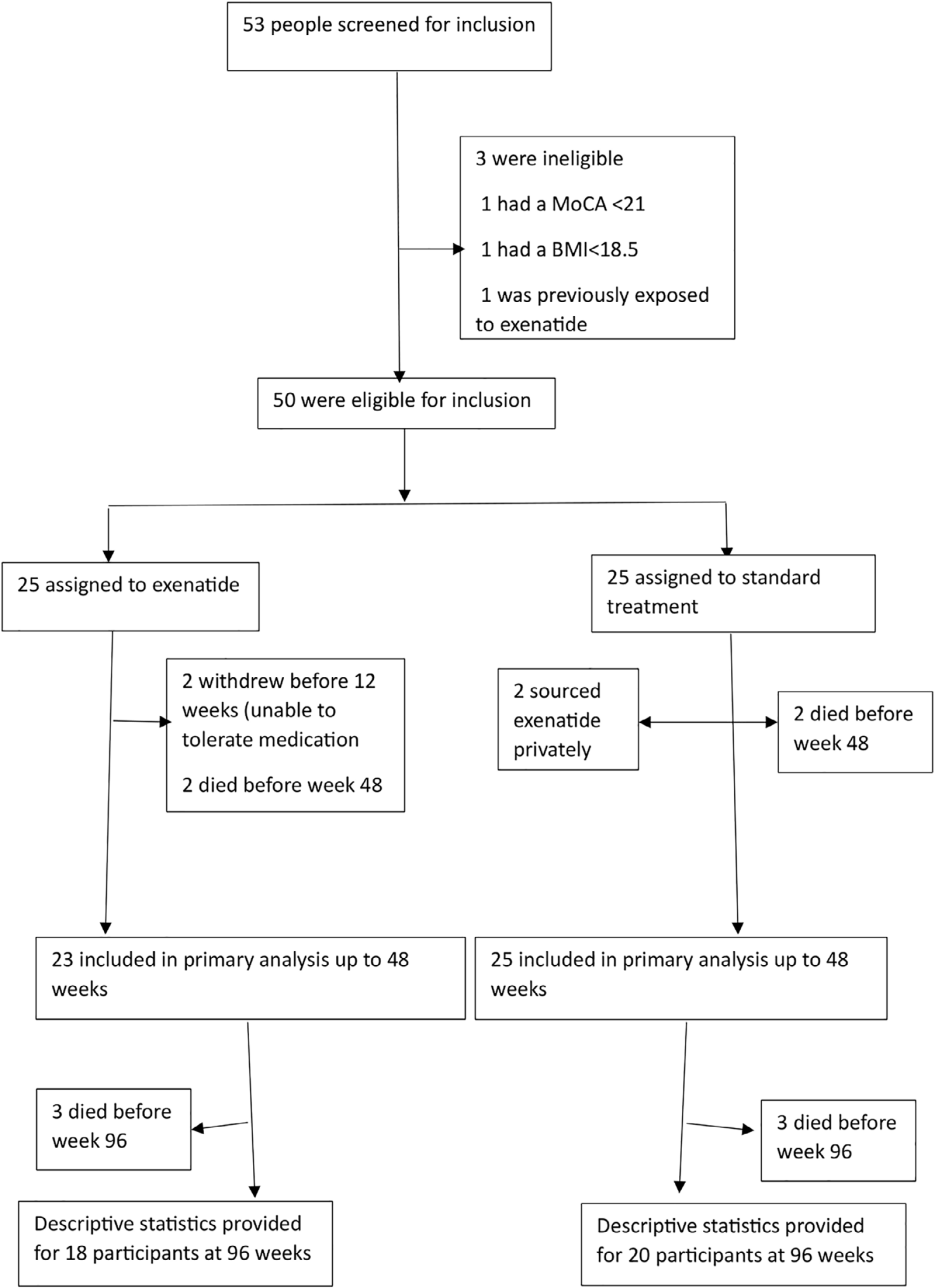
Trial profile.

Between September 23, 2020, and May 6, 2022, 53 participants were screened for eligibility. Fifty participants were randomly assigned to either exenatide or to act as contemporaneous controls (Fig 1). All participants received treatment according to the standard of care for MSA as judged by their primary clinicians. Two people randomized to the control arm sourced exenatide outside of the trial and then used exenatide injections according to the trial protocol for approximately 6 months during the main study period. They had continued follow up as per protocol and were included in the ITT analysis, and also in a CACE analysis, which estimates the effect of treatment for patients that comply with the protocol. Questionnaire responses at each visit suggested that compliance with the study drug among those randomized to exenatide was >87.5% at all timepoints.

Participants allocated to exenatide had higher baseline UMSARS parts I + II scores than those assigned to the control arm (Table 1). Seventy‐two percent (18 of 25) of the exenatide treated group were receiving dopaminergic replacement therapy at baseline, compared with 64% (16 of 25) of the control group.

**TABLE 1:** Baseline Characteristics by Treatment Group Allocation

	Control (N = 25)	Exenatide (N = 25)
Age, yr	62.4 (7.3)	63.3 (8.4)
Symptom duration, yr	4.0 (1.7)	4.0 (1.9)
Duration since diagnosis, yr	0.9 (0.6)	1.2 (1.0)
Sex		
M	12 (48%)	12 (48%)
F	13 (52%)	13 (52%)
Subtype		
MSA‐P	14 (56%)	14 (56%)
MSA‐C	11 (44%)	11 (44%)
Weight, kg	76.7 (15.0)	74.2 (15.4)
UMSARS parts I + II	42.6 (8.2)	46.0 (11.1)
Anti‐parkinsonian medications	11 (44%)	15 (60%)
Orthostatic hypotension medications	2 (8%)	7 (28%)
Ethnicity		
Chinese	1 (4%)	0 (0%)
Indian	0 (0%)	3 (12%)
Pakistani	1 (4%)	0 (0%)
White British	19 (76%)	17 (68%)
White Irish	0 (0%)	1 (4%)
White Other	3 (12%)	3 (12%)
Other	1 (4%)	1 (4%)

*Note*: Numerical data is reported as mean (standard deviation) and categorical data as number (percentage). Anti‐parkinsonian medications were defined as levodopa, dopamine agonists, monoamine oxidase type‐B inhibitors, catechol‐O‐methyltransferase inhibitors, anticholinergics, and amantadine. Anti‐orthostatic hypotension medications included fludrocortisone, midodrine, ephedrine hydrochloride, and pyridostigmine.

MSA‐C = multiple system atrophy cerebellar subtype; MSA‐P = multiple system atrophy parkinsonian subtype; UMSARS = Unified Multiple System Atrophy Rating Scale.

**FIGURE 2:**
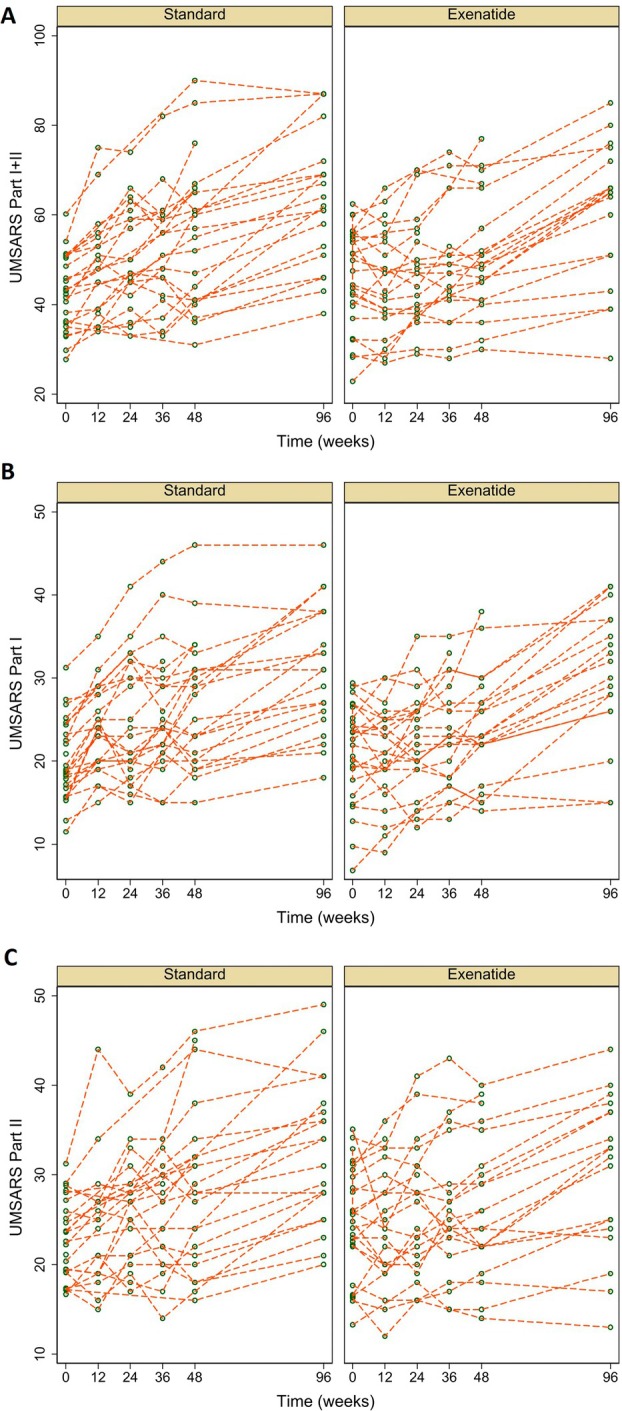
UMSARS over time (baseline, 12, 24, 36, 48, and 96 weeks) line plot, stratified by trial arm. (A) UMSARS parts I + II totals combined. (B) UMSARS part I. (C) UMSARS part II. UMSARS = Unified Multiple System Atrophy Rating Scale. [Color figure can be viewed at www.annalsofneurology.org]

At 48 weeks, UMSARS parts I + II scores had worsened by 13.3 points (95% CI = 9.2 to 17.3, SD = 9.4) in the standard treatment group and by 6.1 points (95% CI = 3.0 to 9.3, SD = 6.9) in the exenatide group; there was a significant adjusted difference of −7.44 points (−11.29 to −3.58) favoring exenatide (*p* = 0·0003). The trajectories for each individual participant are shown in Figure 2. Similar results (and the same conclusions) were obtained after imputing missing data under a variety of assumptions. Both UMSARS part I and UMSARS part II sub‐scores favored exenatide at 48 weeks (Table 2). The results for the CACE analysis was a difference of −8.1 points (−3.3 to −12.9, *p* = 0.001). Alongside this, we noted a significant difference favoring exenatide with higher CGI scores at 48 weeks in exenatide participants compared with the healthy controls (odds ratio [OR] = 4.4, 95% CI = 1.3 to 14.8 *p* = 0·016; Table 3 and Supplementary Table S1).

**TABLE 2:** UMSARS Scores Between Baseline and 96 Weeks

	Baseline	12 weeks	24 weeks	36 weeks	48 weeks	Change* (0–48 weeks)	Difference between Exenatide & Control at 48 weeks (Point estimate, 95% CI, *p* value)	96 weeks	Change* (0–96 weeks)
UMSARS (Part 1 + 2) Mean (SD)									
Exenatide	46.0 (11.1)	45.6 (11.2)	47.7 (12.1)	48.3 (12.7)	50.7 (13.1)	6.1 (6.9)	Coefficient 7.4 (95% CI −11.3, −3.6) *p* < 0.001	60.6 (15.7)	17.1 (9.7)
Control	42.6 (8.2)	48.2 (11.9)	50.6 (11.2)	52.0 (12.4)	55.6 (15.7)	13.3 (9.4)		63.6 (14.8)	22.1 (10.7)
UMSARS Part 1 Mean (SD)									
Exenatide	21.2 (5.9)	20.8 (5.6)	22.2 (5.9)	23.1 (6.2)	24.0 (6.5)	3.3 (3.8)	Coefficient −3.7 (95% CI −6.4, −1.0) *p* = 0.008	30.4 (8.0)	9.9 (5.4)
Control	20.0 (4.8)	23.5 (5.0)	24.7 (7.1)	26.0 (7.1)	27.0 (7.5)	7.2 (4.8)		31.1 (7.6)	12.1 (6.1)
UMSARS Part 2 Mean (SD)									
Exenatide	24.8 (6.3)	24.1 (6.9)	25.8 (7.0)	26.4 (7.3)	26.7 (7.7)	2.8 (4.5)	Coefficient −3.5 (95% CI‐6.7, −0.3) *p* = 0.036	30.2 (8.7)	7.2 (5.8)
Control	22.7 (4.5)	23.8 (6.4)	26.0 (5.6)	25.5 (6.9)	28.6 (8.8)	6.1 (5.8)		32.5 (8.1)	10.1 (5.5)

**TABLE 3:** Changes in Secondary Outcomes Between Baseline and 48 Weeks, and Between Baseline and 96 Weeks

	Base line	12 weeks	24 weeks	36 weeks	48 weeks	Change (0–48 weeks)	Difference between Exenatide & Control at 48 weeks (Ratio, 95% CI, *p* value)	96 weeks	Change (0–96 weeks)	Difference between Exenatide & Control at 96 weeks (Ratio, 95% CI, *p* value)
UMSARS Part IV^a^ Mean (SD)										
Exenatide	2.6 (1.1)	2.5 (0.8)	2.8 (0.9)	2.8 (0.9)	2.8 (1.1)	0.3 (0.9)	OR 0.65, (95% CI 0.19–2.3), *p* = 0.52	3.3 (1.2)	0.9 (0.6)	OR 0.63 (95% CI 0.16, 2.5), *p* = 0.51
Control	2.4 (0.9)	2.7 (0.9)	3.0 (1.0)	3.0 (1.1)	3.0 (0.9)	0.6 (0.6)		3.6 (0.9)	1.3 (0.6)	
CGI^b^ Mean (SD)										
Exenatide	‐	‐	‐	‐	3.1 (1.0)	‐	OR 4.4 (95% CI 1.3–14.8), *p* = 0.016	2.1 (0.8)	‐	OR 1.25 (0.38, 4.2) *p* = 0.72
Control	‐	‐	‐	‐	2.4 (0.8)	‐		2.0 (0.7)	‐	
UMSARS‐I item 7 (Independent Ambulation score = 4), N (%)										
Exenatide	0 (0%)	0 (0%)	1 (4%)	2 (8%)	2 (8%)	‐	HR 1.04 (95% CI 0.1–7.4) *p* = 0.97	9 (36%)	‐	HR 1.36 (95% CI 0.51, 3.67) *p* = 0.54
Control	0 (0%)	0 (0%)	2 (8%)	2 (8%)	2 (8%)	‐		7 (28%)	‐	
UMSARS‐I item 1, Speech (≥3)^c^ N (%)										
Exenatide	4 (16%)	2 (9%)	3 (13%)	3 (13%)	3 (14%)	‐	OR 0.35, (95% CI 0.07–1.8), *p* = 0.21	15 (75%)	‐	OR 0.90 (0.21, 3.96) *p* = 0.90
Control	3 (12%)	2 (8%)	3 (13%)	4 (18%)	7 (30%)	‐		13 (72%)	‐	
UMSARS‐I item 2, Swallowing (≥3)^c^ N (%)										
Exenatide	0 (0%)	3 (13%)	0 (0%)	2 (9%)	2 (10%)	‐	OR 0.23 (95% CI 0.04, 1.3) *p* = 0.10	6 (33%)	‐	OR 0.65 (0.17, 2.48) *p* = 0.53
Control	2 (8%)	4 (17%)	5 (21%)	12 (50%)	8 (35%)	‐		9 (45%)	‐	
UMSARS‐I item 8, Falling (≥3)^c^ N (%)										
Exenatide	6 (24%)	2 (8.3%)	2 (8.3%)	4 (18.2%)	3 (14.3%)	‐	OR 0.17 (95% CI 0.02–1.7) *p* = 0.13	9 (50%)	‐	OR 0.69 (0.16, 3.04) *p* = 0.62
Control	3 (12%)	5 (21.8%)	5 (20.8%)	6 (25%)	6 (26.1%)	‐		11 (55%)	‐	

CI = confidence interval; OR = odds ratio; UMSARS = unified multiple system atrophy rating scale.

^a^ Ordinal response, interpreted as numerical.

^b^ Is a change from baseline in itself, therefore no change reported.

^c^ Patients scoring **≥**3 at screening were excluded from participation, however baseline assessments were performed post randomisation hence a small percentage of participants score **≥**3 at baseline.

In contrast, we noted no significant differences between the exenatide and control groups in the UMSARS part IV proportion of participants with loss of independent ambulation defined by a score of 4 or more in UMSARS part I item 7 (walking), proportion of participants reaching a score of 3 or more on UMSARS part I items 1 (speech), 2 (swallowing), and 8 (falling), MSA‐QOL scale, number of falls, BDI‐II, or MoCA scores (see Table 3). There were no significant differences in UMSARS part III vital signs with the exception that the standing diastolic blood pressure reduced more between baseline and 48 weeks with exenatide treatment (difference 6.9 mmHg, 95% CI = −13.3 to −0.3, *p* = 0·04), and no difference in the frequency of anti‐parkinsonian or anti‐orthostatic hypotension drug use between groups (OR = 0.4, 95% CI = 0.05 to 3.0, *p* = 0.47).

At 96 weeks, UMSARS parts I + II scores had deteriorated by 22.1 points in the control group compared to baseline and those in the exenatide group by 17.1 points (see Table 2). There was no longer any advantage in the CGI scores. At 96 weeks, MoCA scores were lower in the exenatide treated participants.

All participants in each group were able to complete a timed walk at baseline. In the exenatide group, 17 of 21 (81%) participants were able to complete a timed walk at 48 weeks compared with 18 of 23 (78%) participants allocated to the control arm. For testing purposes, we imputed maximum values for those participants unable to walk. There was no significant difference between the groups in the time taken (*p* value: on medication = 0.680 and off medication = 0.521), nor the number of steps required to complete the walk at 48 weeks (*p*‐value: on medication = 0.729 and off medication = 0.914). Forty‐four percent (8 of 18) of the exenatide treated group assessed at 96 weeks had become non‐ambulant compared with 35% (7 of 20) of the control participants.

The mean serum exenatide level among participants compliant with injections at 48 weeks was 1791 pg/ml (SD = 905). This excludes 2 individuals with very high levels detected, likely due to the presence of anti‐exenatide antibody formation which cross reacts with the assay. Thirty‐nine individuals had successful CSF collection at baseline, of which 23 had a repeat lumbar puncture at 48 weeks. The mean CSF exenatide level among participants compliant with injections was 16.9 pg/ml (SD = 12.5).

**FIGURE 3:**
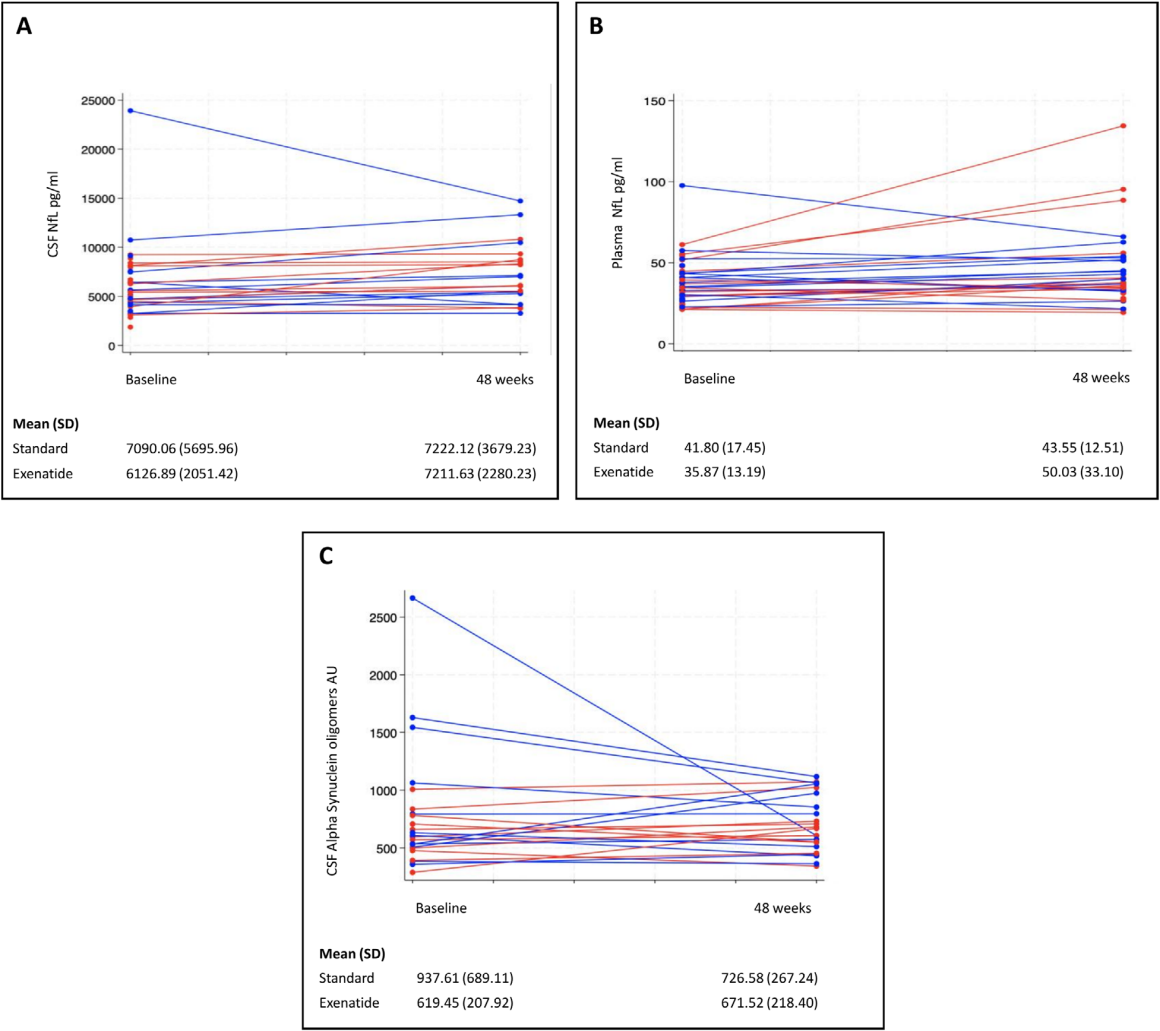
(A) Individual changes in CSF NfL between baseline and week 48. (B) Individual changes in plasma NfL between baseline and week 48. (C) Individual changes in CSF alpha synuclein oligomers between baseline and week 48. Blue and red lines represent the standard of care (N = 16 for plasma and N = 12 for CSF) and exenatide groups (N = 14 for plasma and N = 11 for CSF), respectively. CSF = cerebrospinal fluid; NfL = neurofilament light. [Color figure can be viewed at www.annalsofneurology.org]

Compared to baseline, there was a greater nonsignificant increase in both plasma and CSF NfL in the exenatide treated group compared with the healthy controls by week 48, but these changes were largely driven by 1 or 2 outliers (Fig 3A, B). There was no significant difference in the change in alpha‐synuclein oligomer load in CSF between groups (Chi2 = 1.83, *p* = 0.17; Fig 3C).

**FIGURE 4:**
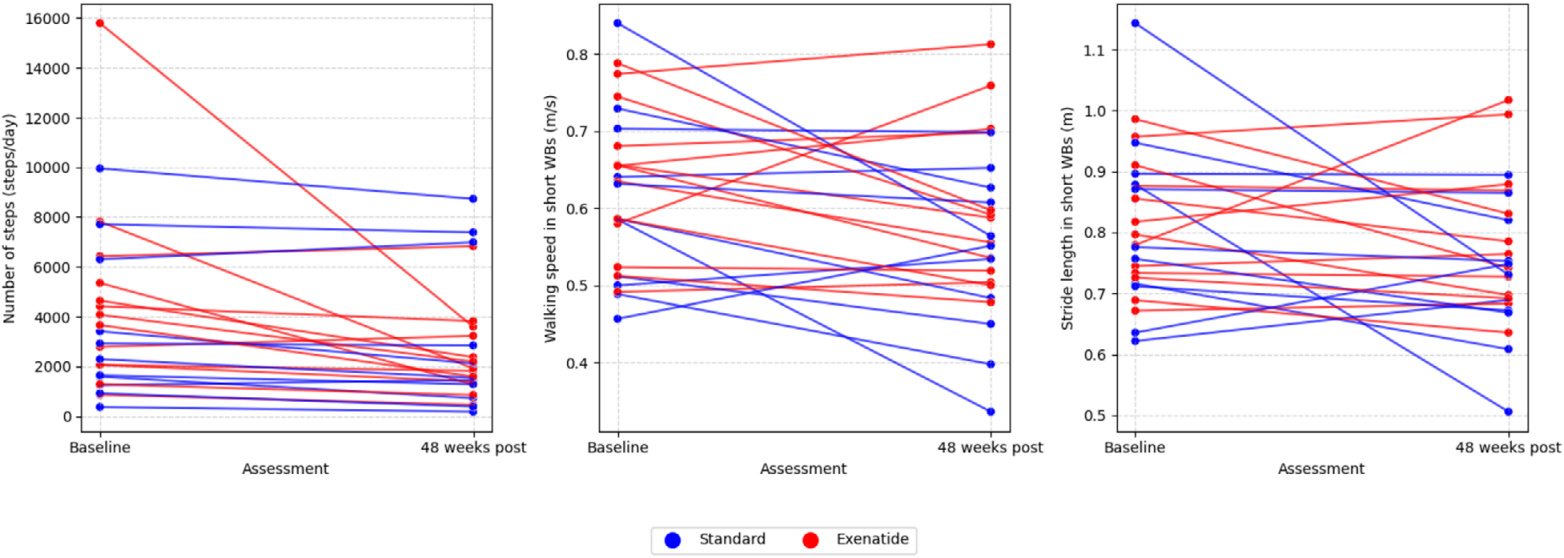
Changes in digital mobility outcomes between assessments at baseline and week 48 for the standard and exenatide trial arms. [Color figure can be viewed at www.annalsofneurology.org]

Twenty‐four participants had valid Axivity data at both baseline and 48 weeks (Supplementary Table S2). The digital mobility outcomes of interest did not differ between treatment groups over 48 weeks. Daily step count declined by 667 steps/day in the exenatide group and 360 steps/day in the control arm (*p* = 0.124), but this change in the active group was largely driven by an outlier (Fig 4). Walking speed and stride length showed no difference in the exenatide group versus the control group.

There were no differences between any of the MRI volume/tissue comparisons according to randomization that surpassed even very lenient thresholds (*p* < 0·001 uncorrected) or small volume analyses.

The frequency of adverse effects did not differ between groups (Table 4). Two participants randomized to exenatide, withdrew because they were unable to tolerate the injections (mainly due to a worsening in postural hypotension in one participant and significant nausea in the other). Weight loss occurred in both groups, but was more common in the exenatide participants (see Table 3). Weight changes did not contribute to mortality when accounting for treatment allocation and MSA sub‐type (hazard ratio [HR] = 1.02, 95% CI = 0.98 to 1.06, *p* = 0.27). Thirty‐nine serious adverse events were recorded, 23 in the exenatide group and 18 in the control group. Two people died from each group during the 48 weeks exposure period and a total of 5 people died in each treatment arm by 96 weeks (see Fig 1). None of the serious adverse events or deaths were judged to be related to the study intervention.

**TABLE 4:** Adverse Events

Adverse Events	Exenatide	Standard of Care
Gastrointestinal disorders, for example, nausea, bloating, and constipation	36 (n = 14)	17 (n = 12)
Skin disorders, for example, nodules and rashes	14 (n = 12)	5 (n = 4)
Infections, for example, UTI and chest	38 (n = 16)	16 (n = 14)
Serum amylase increase	1	1
Weight loss, anorexia	14 (n = 11)	4 (n = 4)
General, for example, fatigue and pain	8 (n = 6)	6 (n = 6)
Gait disturbance	19 (n = 17)	19 (n = 17)
Other miscellaneous	64 (n = 21)	52 (n = 22)
**Total**	**194**	**120**
Serious adverse events		
Infections	9 (n = 6)	8 (n = 7)
Gastrointestinal	2 (n = 2)	4 (n = 4)
Cardiac	0	1
Falls	2 (n = 2)	0
Worsening MSA	2 (n = 2)	1
Anxiety and depression	2 (n = 1)	0
Other	4 (n = 3)	4 (n = 4)
Serum amylase increase	2 (n = 1)	0
**Total**	**23**	**18**

*Note*: Data are presented as the number of events (number of participants).

MSA = multiple system atrophy; UTI = urinary tract infection.

## Discussion

We report the results of a small, open label exploratory trial, designed to provide proof‐of‐concept data to assess whether exenatide might have disease modifying properties among participants with MSA. We found an advantage among the group randomly assigned to self‐administer exenatide based on both UMSARS part I (participant symptom severity), UMSARS part II (clinician rating of severity), and the combination of these measures. A small early improvement in UMSARS scores was detectable at 12 weeks in the exenatide group, suggesting either a placebo effect or that the drug may have symptomatic effects.^13^ Furthermore, the exenatide group declined by 6.1 points whereas the control group declined by 13.3 points per 48 weeks. This is in keeping with previous prospective natural history studies of European populations,^33^ although the rate of decline in the standard group is almost double the expected rate based on the MSA‐Ras trial.^30^ This discrepancy could potentially be explained by the MSA‐Ras trial having a lower disease severity at baseline compared with the participants in our cohort.

We were acutely aware that the open label design would make the trial vulnerable to placebo effects and/or observer bias, and therefore deliberately included a long washout period to help (1) distinguish symptomatic from disease modifying effects and (2) allow sufficient time for potential placebo effects to lessen. We also predefined for analysis those secondary outcomes that could be considered as more objective measures of disease progression. However, we did not find an advantage of exenatide exposure on any measure, including falls, bulbar impairment, mortality, gait activity, or MRI regional volumes. This was also true for changes in NfL or CSF alpha‐synuclein oligomer levels, although none of these biomarkers have been validated as sensitive predictors of disease progression over time.^34^

The absence of any of the objective data favoring exenatide casts doubt on the likely benefits of this intervention that are indicated by the UMSARS scores. Accordingly, we have to clearly acknowledge that the advantage seen in the primary outcome may be entirely due to placebo effects and/or observer bias. Therefore, and despite the absence of any proven disease modifying interventions for MSA, we do not believe that the data from this randomized trial support current use of this drug in patients with MSA. These results must also be viewed in the context of a recently published phase III trial evaluating exenatide as a potential disease modifying treatment for PD. Whereas earlier smaller trials had shown positive effects, the larger, longer term trial found no advantage of exenatide in PD.^35^

Our trial population included participants with both MSA‐P and MSA‐C, and the randomization processes ensured that these subtypes were perfectly balanced between treatment groups. We did not find evidence to indicate any advantage in any of the objective measures according to disease subtype. It remains of great interest whether there may be a subgroup of people affected by MSA in whom exenatide has objective evidence of benefit and further post hoc analyses are planned but these may be underpowered to detect changes in smaller numbers of participants.

The drug was generally well tolerated, and participants reported the well‐recognized adverse events associated with exenatide (eg, gastrointestinal disorders). Such problems were transient and settled after 6 to 8 weeks on treatment. An average weight loss of 5 kg in the exenatide group was observed, with all participants remaining in a healthy BMI range. There was no evidence that weight loss influenced survival in our cohort.

High levels of self‐reported compliance are mirrored by the serum levels of exenatide detected. Only 1% of the serum exenatide level was detectable in CSF, which confirms that the drug can cross the blood brain barrier and was comparable with previous studies.^35^ It is unclear what level of CNS penetration may be necessary for a GLP‐1 RA to exhibit the neuroprotective effects seen in the laboratory. The low level of exenatide detected in the CSF may not accurately reflect neuroparenchymal levels, although evidence from a previous exenatide trial in PD suggests this level of central nervous system (CNS) penetration was sufficient to engage neuronal insulin resistance pathways,^12^ but casts doubt whether this agent can sufficiently engage neuronal or glial GLP‐1 receptors that are theoretically necessary. Further target engagement work evaluating changes in the insulin signaling pathway in neuronal/glial exosomes may help address this uncertainty in MSA.

### Limitations

The monocentric open label design limits our interpretation of the results, although our aim was to provide the proof‐of‐concept data that would justify a larger placebo‐controlled randomized clinical trial in MSA. Further, we included participants who were within 5 years from symptom onset to ensure that we were able to meet our recruitment targets. It could be argued that such a broad disease duration may have included individuals with fairly advanced MSA who were less likely to benefit from disease modifying treatments. However, the disease duration from precise MSA diagnosis was approximately 1 year in our cohort.

Two individuals in the control arm were disappointed by the randomization outcome and managed to secure supplies of exenatide privately, which they readily admitted to the trial team. There was, however, no fundamental difference in any of the results irrespective of whether our analyses used ITT or complier average causal effect analyses to adjust for these protocol deviations.

The MRI analysis between treatment arms was small, with only 7 participants in each arm with complete longitudinal data. These high dropout rates are similar to other longitudinal MRI studies in MSA^36^ and likely reflects the aggressive nature of MSA coupled with the difficulties in getting individuals comfortably inside the scanner due to worsening postural deformities. Consequently, the MRI analysis may not have had enough power to detect subtle changes in regional brain volume. However, the voxel‐wise methods used, that use segmentation pipelines specifically optimized for brainstem structures, are more sensitive compared with standard approaches, and coupled with the negative results from other independent biomarkers, most likely reflect a lack of treatment effect.

There remains a great deal of interest in the potential role of GLP‐1 RA, or dual/triple agonists as treatments for neurodegenerative diseases.^37^, ^38^ There remain significant major issues that need to be further resolved, however, in terms of degree of CNS penetration between members of this class of drug, confirmation of target engagement and identifying potential subgroups within the different neurodegenerative diseases who might be most likely to respond.

## Author Contributions

**Nirosen Vijiaratnam:** Investigation; writing – original draft. **Christine Girges:** Writing – original draft; investigation. **Martin Wiegand:** Formal analysis; data curation. **Claudia Ismail:** Investigation; project administration. **Alexandra Lameirinhas:** Investigation. **Alison Yarnall:** Investigation; writing – original draft. **Cameron Kirk:** Writing – original draft; investigation. **Silvia Del‐Din:** Writing – original draft; investigation. **Lynn Rochester:** Investigation. **Christopher Kobylecki:** Supervision. **Gareth Ambler:** Formal analysis; data curation. **Simon Skene:** Supervision. **Henry Houlden:** Investigation; conceptualization. **Viorica Chelban:** Investigation; conceptualization. **Amanda Heslegrave:** Investigation. **Wendy Phillips:** Supervision. **Alan Whone:** Supervision. **Niall Quinn:** Supervision. **Christian Lambert:** Investigation; writing – original draft. **Charlotte Dore:** Investigation. **Huw R. Morris:** Conceptualization. **Mathew H. Horrocks:** Investigation; writing – original draft. **Ji Eun Lee:** Investigation. **Judi O'Shaughnessy:** Investigation. **Yazhou Li:** Investigation. **Nigel H. Greig:** Investigation; writing – review and editing. **Sonia Gandhi:** Investigation; writing – original draft. **Vincenzo Libri:** Methodology; investigation; project administration. **Dilan Athauda:** Investigation; conceptualization; writing – original draft. **Tom Foltynie:** Conceptualization; writing – original draft; writing – review and editing; methodology; funding acquisition.

## Potential Conflicts of Interest

Nothing to report.

## Supporting information


**Table S1.** Further predefined secondary outcomes between Baseline and 48 weeks and between Baseline and 96 weeks.

**Table S2.** Changes from baseline to 48 weeks post of digital mobility outcomes measuring walking activity for exenatide and control trial arms. Changes from baseline were statistically compared between trial arms with appropriate parametric (*t* test) or non‐parametric (Kruskal‐Wallis) method.

## Data Availability

Trial data will be available for sharing by the chief investigator (author T.F.) on reasonable request.
