# Supplementary material for: Exenatide Once Weekly in the Treatment of Patients with Multiple System Atrophy
Source: Ann Neurol. 2025 Jul 29;98(5):991–1003. doi: 10.1002/ana.70004 (PMC12577669; doi:10.1002/ana.70004)
Supplement: Supplementary file 1 — Table S1. Further predefined secondary outcomes between Baseline and 48 weeks and between Baseline and 96 weeks. [file ANA-98-991-s001.docx]

Supplementary Table 1. Further predefined secondary outcomes between Baseline and 48 weeks and between Baseline and 96 weeks.

|  | Base  line | 12 wks | 24 wks | 36 wks | 48  wks | Change  (0-48 wks) | Difference between Exenatide & Control at 48 weeks  (Ratio, 95%CI, p value) | 96 wks | Change  (0-96 weeks) |
| --- | --- | --- | --- | --- | --- | --- | --- | --- | --- |
| **MSA-QoL Motor Domain**  **Mean (SD)** | | | | | | | | | |
| Exenatide | 44.2 (16.3) | - | - | - | 53.1 (19.8) | 9.5 (14.5) | Coefficient -0.1, (CI -9.2, 9.1), p=0.99 | 65.9 (24.4) | 22.3 (19.9) |
| Control | 42.4 (16.0) | - | - | - | 53.7 (23.7) | 10.4 (15.7) |  | 65.6 (24.3) | 24.4 (13.9) |
| **MSA-QoL Non-motor Domain**  **Mean (SD)** | | | | | | | | | |
| Exenatide | 36.3 (17.9) | - | - | - | 37.4 (17.8) | 3.3 (16.3) | Coefficient -0.5, (CI -9.0, 7.9), p=0.90 | 43.4 (19.5) | 9.8 (14.3) |
| Control | 36.0 (13.6) | - | - | - | 39.3 (15.4) | 3.4 (13.0) |  | 43.4 (18.0) | 8.6 (15.9) |
| **MSA-QoL Emotional/Social Functioning Domain**  **Mean (SD)** | | | | | | | | | |
| Exenatide | 31.0 (22.0) | - | - | - | 33.3 (22.8) | 5.4 (22.6) | Coefficient -3.8, (CI -15.6, 8.0), p=0.52 | 44.4 (27.6) | 13.5 (19.9) |
| Control | 35.2 (23.7) | - | - | - | 43.1 (23.9) | 6.0 (17.5) |  | 50.0 (29.8) | 14.0 (20.2) |
| **Beck depression inventory**  **Mean (SD)** | | | | | | | | | |
| Exenatide | 12.5 (6.2) | 17.9 (13.1) | 17.0 (11.1) | 14.0 (9.7) | 14.3 (8.8) | 2.4 (4.9) | Coefficient 1.02, (95% CI -3.0, 5.1) p=0.61 | 17.9 (13.1) | 6.3 (9.4) |
| Control | 14.0 (7.9) | 17.2 (11.2) | 15.3 (10.1) | 16.8 (8.9) | 15.2 (8.0) | 0.9 (7.9) |  | 17.2 (11.2) | 3.1 (11.3) |
| **MOCA**  **Mean (SD)** | | | | | | | | | |
| Exenatide | 26.5 (2.6) | 26.0 (2.5) | 26.4 (3.6) | 26.1 (3.6) | 26.0 (2.7) | -0.4 (2.0) | -0.07 points (95% CI -1.3, 1.2) p=0.91 | 24.8 (4.1) | -2.3 (3.5) |
| Control | 27.8 (1.5) | 27.1 (1.6) | 27.8 (1.8) | 27.3 (1.8) | 27.2 (2.5) | -0.6 (1.9) |  | 27.7 (1.4) | -0.4 (1.5) |
| **Number of falls**  **Mean (SD)** | | | | | | | | | |
| Exenatide | 1.9 (2.8) | 1.8 (2.6) | 1.1 (1.6) | 1.1 (2.5) | 1.7 (2.9) | -0.4 (3.4) | Coefficient -0.34 (95% CI -1.4, 0.7) p=0.53 | 2.1 (7.2) | -0.4 (7.3) |
| Control | 1.5 (1.7) | 3.5 (6.4) | 1.6 (2.8) | 1.2 (2.0) | 3.0 (6.8) | 1.5 (5.9) |  | 2.5 (5.7) | 1.1 (4.9) |
| **Weight (Kg)**  **Mean (SD)** | | | | | | | | | |
| Exenatide | 74.2 (15.4) | 72.4 (16.5) | 68.1 (14.0) | 69.6 (15.8) | 68.2 (15.2) | -5.0 (7.2) |  | 71.0 (17.7) | -2.8 (9.6) |
| Control | 76.7 (15.0) | 74.3 (16.2) | 74.4 (15.2) | 73.9 (16.2) | 72.8 (15.1) | -0.2 (4.9) |  | 75.4 (17.0) | -1.0 (7.7) |
| **LEDD (mg)**  **Mean (SD)** | | | | | | | | | |
| Exenatide | 426.9 (443.7) |  |  |  | 342.9 (404.4) | -23.4 (106.4) | Coefficient -15.2 (95% CI -199.7,169.3) p=0.869 | 346.8  (462.9) | 13.7  (111.5) |
| Control | 324.4 (400.5) |  |  |  | 356.5 (437.6) | 19.0 (446.2) |  | 358.1  (474.1) | 28.3  (467.6) |
| **Number of Steps (Off Medication)**  **Median [IQR]** | | | | | | | | | |
| Exenatide | 74  [43, 94] |  |  |  | 81  [57, 100] | 16 [6,23] | Mann-Whitney median comparison p = 0.914 | - | - |
| Control | 52  [32.5, 75] |  |  |  | 65.5  [43,86.5] | 25.5 [1.5,39] |  | - | - |
| **Number of Steps (On Medication)**  **Median [IQR]** | | | | | | | | | |
| Exenatide | 46  [33, 52] |  |  |  | 65  [32, 89] | 11.5 [4,23] | Mann-Whitney median comparison p = 0.729 | - | - |
| Control | 32  [28, 45] |  |  |  | 43  [34, 66] | 15.5 [9,26] |  | - | - |
| **Time Taken (Off Medication)**  **Median [IQR]** | | | | | | | | | |
| Exenatide | 67  [32.7,110.7] |  |  |  | 56.9 [39.2, 98] | 8.9 [0.3,20.8] | Mann-Whitney median comparison p = 0.521 | - | - |
| Control | 61.95  [35, 126] |  |  |  | 61.2 [39.8,125] | 0.8  [-824.5,  46.8] |  | - | - |
| **Time Taken (On Medication)**  **Median [IQR]** | | | | | | | | | |
| Exenatide | 40.5  [31, 69] |  |  |  | 42  [28, 61] | 4 [2,18] | Mann-Whitney median comparison p = 0.680 | - | - |
| Control | 108  [32, 184.5] |  |  |  | 37  [28, 66] | -157  [-157, -157] |  | - | - |
