# Supplementary material for: Exenatide Once Weekly in the Treatment of Patients with Multiple System Atrophy
Source: Ann Neurol. 2025 Jul 29;98(5):991–1003. doi: 10.1002/ana.70004 (PMC12577669; doi:10.1002/ana.70004)
Supplement: Supplementary file 2 — Table S2. Changes from baseline to 48 weeks post of digital mobility outcomes measuring walking activity for exenatide and control trial arms. Changes from baseline were statistically compared between trial arms with appropriate parametric (t test) or non‐parametric (Kruskal‐Wallis) method. [file ANA-98-991-s002.docx]

**Supplementary table 2.** Changes from baseline to 48 weeks-post of digital mobility outcomes measuring walking activity for exenatide and control trial arms. Change from baseline were statistically compared between trial arms with appropriate parametric (t-test) or non-parametric (Kruskal-Wallis) method.

| **Walking activity** | **Exenatide (n = 13)** | |  | **Control group (n = 11)** | |  |  |
| --- | --- | --- | --- | --- | --- | --- | --- |
| **Volume** | **Baseline** | **48-Weeks** | **Change from baseline to 48 weeks** | **Baseline** | **48-Weeks** | **Change from baseline to 48 weeks** | **Comparison of change from baseline between trial arms** |
| Number of steps (steps/day) | 4078.8 [2060.8,5345.8]  4709.4 (3891.7) | 1919.9 [1394.0,3227.8]  2410.8 (1669.7) | -666.8  [-2263.5,-406.6]  2298.6 (3486.9) | 2292.5 [1415.8,4854.0]  3486.1 (3124.9) | 1541.4 [1003.2,4914.1]  3056.7 (3100.2) | -360.4  ([-807.4,-133.0]  -429.4 (594.2) | 0.124 |
| Walking duration (minutes/day) | 47.1  [25.7,53.6]  47.7 (33.3) | 24.0  [17.2,36.2]  27.5 (16.5) | -8.5  [-23.1,-3.3]  -20.1 (28.1) | 23.9  [16.8,53.]  37.9 (31.8) | 18.1  [11.7,52.5]  33.1 (31.6) | -4.5  [-8.8,-1.7]  -4.8 (7.7) | 0.111 |
| **Pattern** |  |  |  |  |  |  |  |
| Number of WB (WB/day) | 187.7 [125.0,304.9]  203.7 (100.4) | 115.9 [96.4,191.2]  142.6 (78.7) | -34.1  [-115.0,-8.1]  -61.0 (73.3) | 113.4 [97.6,205.6]  154.0 (83.1) | 116.0 [72.1,190.8]  130.6 (91.0) | -23.0  [-46.3,-7.2]  -23.4 (45.8) | 0.141 |
| Number of WB  > 10s (WB/day) | 64.8  [40.8,89.2]  67.0 (30.6) | 42.4  [30.2,50.0]  67.0 (30.6) | -10.8  [-31.6,-7.4]  -20.8 (23.7) | 47.1  [29.4,77.8]  53.4 (30.4) | 34.5  [21.1,59.9]  44.6 (32.8) | -10.4  [-17.5,-1.4]  -8.8 (16.7) | 0.163 |
| Number of WB  > 30s (WB/day) | 9.8  [6.1,11.9]  10.9 (8.9) | 5.0  [3.4,7.4]  6.5 (5.6) | -4.3  [-6.9,-0.2]  -4.5 (8.8) | 5.1  [4.1,16.8]  10.0 (9.2) | 4.9  [2.1,15.5]  8.8 (9.1) | -1.2  [-2.6,-0.1]  -1.3 (1.5) | 0.104 |
| WB duration (s) | 7.2  [6.9,7.4]  7.4 (0.9) | 7.1  [6.8,8.1]  7.4 (0.9) | -0.3  [-0.5,0.9]  0.0 (1.1) | 7.6  [7.2,8.0]  7.7 (0.9) | 7.5  [7.2,8.2]  7.9 (1.1) | 0.2  [-0.3,0.8]  0.2 (1.4) | 0.736 |
| Maximum WB duration (s) | 23.1  [17.4,32.0]  24.6 (8.6) | 18.7  [16.3,21.0]  21.0 (8.1) | -2.1  [-9.9,1.9]  -3.6 (10.7) | 19.1  [17.1,29.3]  22.8 (6.7) | 24.5  [18.1,25.9]  24.1 (9.6) | 0.1  [-3.6,3.0]  1.3 (7.6) | 0.204 |
| **WB duration variability (COV, %)** | **163.3 [125.3,290.6]**  **185.9 (95.4)** | **101.0 [82.7,152.3]**  **122.4 (57.1)** | **-45.1**  **[-75.3,-28.6]**  **-63.5 (56.5)** | **130.5 [92.6,215.8]**  **159.2 (94.9)** | **109.4 [90.5,233.8]**  **160.5 (98.5)** | **6.8**  **[-20.4,18.2]**  **1.3 (35.9)** | **0.002** |

Data presented as median [25^th^ and 75^th^ percentiles] and mean (SD). WB = Walking bout. Significant results in bold.

**Supplementary file #.**

Over the 48-week trial, only walking bout duration variability changed significantly between treatment arms. Participants receiving exenatide had a greater reduction in the variability in the duration of walking bouts, even after excluding two outliers from the exenatide group. The clinical significance of this is unclear. It should be noted that the comparisons are limited by relatively small numbers within each group and lack of correction for multiple comparisons.

**Supplementary table 2.** Changes from baseline to 48 weeks-post of digital mobility outcomes measuring gait for exenatide and placebo trial arms. Change from baseline were statistically compared between trial arms with appropriate parametric (t-test) or non-parametric (Kruskal-Wallis) method.

| Gait | | **Exenatide (n = 13)** | | **Placebo group (n = 11)** | |  | |
| --- | --- | --- | --- | --- | --- | --- | --- |
|  | **Baseline** | **48-Weeks** | **Change from baseline to 48 weeks** | **Baseline** | **48-Weeks** | **Change from baseline to 48 weeks** | **Comparison of change from baseline between trial arms** |
| **Pace** | | | | | | | |
| Walking speed in shorter  (10-30s) WB (m/s) | 0.65  [0.58, 0.68]  0.64 (0.10) | 0.59  [0.52, 0.70]  0.60 (0.11) | -0.03  [-0.09, 0.02]  -0.03 (0.10) | 0.59  [0.51, 0.67]  0.61 (0.12) | 0.55  [0.47, 0.62]  0.54 (0.11) | -0.06  [-0.10, 0.00]  -0.07 (0.11) | 0.417 |
| Walking speed in longer (>30s) WB (m/s) | 0.71  [0.61, 0.86]  0.74 (0.18) | 0.66  [0.63, 0.75]  0.69 (0.13) | -0.04  [-0.09, 0.01]  -0.06 (0.19) | 0.63  [0.53, 0.80]  0.66 (0.16) | 0.56  [0.52, 0.76]  0.64 (0.15) | -0.03  [-0.09, 0.06]  -0.02 (0.16) | 0.570 |
| Maximum walking speed in WB > 10s (m/s) | 0.82  [0.73, 0.90]  0.84 (0.17) | 0.73  [0.70, 0.82]  0.77 (0.15) | -0.01  [-0.18, 0.02]  -0.07 (0.16) | 0.71  [0.64, 0.95]  0.78 (0.18) | 0.65  [0.59, 0.89]  0.70 (0.19) | -0.11  [-0.15, 0.01]  -0.08 (0.13) | 0.780 |
| Maximum walking speed in longer (>30s) WB (m/s) | 0.78  [0.71, 1.02]  0.86 (0.23) | 0.78  [0.69, 0.90]  0.80 (0.21) | -0.05  [-0.15, 0.00]  -0.06 (0.27) | 0.74  [0.58, 1.01]  0.78 (0.24) | 0.63  [0.56, 0.95]  0.74 (0.21) | -0.08  [-0.18, 0.04]  -0.04 (0.18) | 0.804 |
| Stride length in shorter  (10-30s) WB (m), | 0.80  [0.73, 0.88]  0.81 (0.10) | 0.76  [0.70, 0.87]  0.79 (0.12) | -0.01  [-0.07, 0.02]  -0.02 (0.10) | 0.78  [0.71, 0.89]  0.81 (0.15) | 0.73  [0.67, 0.79]  0.72 (0.11) | -0.04  [-0.12, -0.00]  -0.09 (0.17) | 0.400 |
| Stride length in longer  (>30s) WB (cm), | 0.93  [0.78, 0.97]  0.93 (0.18) | 0.87  [0.81, 0.91]  0.90 (0.15) | -0.05  [-0.09, -0.00]  -0.03 (0.23) | 0.87  [0.71, 0.99]  0.86 (0.20) | 0.81  [0.72, 0.95]  0.83 (0.15) | -0.03  [-0.06, 0.05]  -0.03 (0.23) | 0.505 |
| **Rhythm** | | | | | | | |
| Cadence in all WB (steps/min) | 94.22  [90.83, 97.11]  94.33 (3.89) | 91.25  [89.16, 93.21]  91.69 (3.63) | -1.91  [-5.72, 0.26]  -2.64 (3.81) | 89.88  [85.32, 95.37]  90.22 (6.04) | 89.46  [86.40, 93.84]  89.78 (5.20) | 0.08  [-1.07, 0.94]  -0.44 (3.02) | 0.173 |
| Cadence in longer  (>30s) WB (steps/min) | 94.58  [91.68, 97.94]  95.27 (5.92) | 90.43  [89.30, 95.43]  91.41 (6.11) | -0.58  [-7.51, 0.50]  -3.86 (7.19) | 93.13  [84.54, 98.53]  91.81 (8.17) | 90.16  [86.94, 97.48]  91.90 (7.38) | 1.32  [-2.08, 2.94]  0.09 (6.92) | 0.111 |
| Maximum cadence in longer (>30s) WB (steps/min) | 102.66  [96.78, 113.27]  103.94 (8.06) | 100.36  [92.56, 104.18]  99.38 (7.85) | -4.44  [-10.07, -0.62]  -4.56 (8.15) | 105.82  [89.79, 107.97]  99.96 (11.42) | 94.47  [88.94, 106.87]  97.99 (10.33) | -0.01  [-4.44, 2.98]  -1.97 (8.28) | 0.449 |
| Stride duration in all WB (s) | 1.15  [1.13, 1.17]  1.15 (0.06) | 1.15  [1.13, 1.20]  1.16 (0.05) | -0.00  [-0.03, 0.03]  0.01 (0.09) | 1.20  [1.11, 1.28]  1.20 (0.11) | 1.16  [1.12, 1.19]  1.16 (0.05) | -0.01  [-0.09, 0.01]  -0.04 (0.09) | 0.339 |
| Stride duration in longer (>30s) WB (s) | 1.17  [1.12, 1.21]  1.17 (0.09) | 1.18  [1.16, 1.23]  1.18 (0.07) | 0.03  [-0.05, 0.07]  0.01 (0.10) | 1.17  [1.08, 1.22]  1.15 (0.13) | 1.14  [1.09, 1.22]  1.14 (0.10) | 0.02  [-0.04, 0.07]  -0.01 (0.12) | 0.794 |

Data presented as median [25^th^ and 75^th^ percentiles] and mean (SD). WB = Walking bout.
